# Structural and developmental insights into the muscles involved in lionfish (*Pterois* spp.) vocalisations

**DOI:** 10.1111/jfb.70183

**Published:** 2025-08-28

**Authors:** Roxanne B. Holmes, Nadia M. Hamilton, Katharine E. Criswell, Keturah Z. Smithson, James E. Herbert‐Read, Lucille Chapuis

**Affiliations:** ^1^ Department of Zoology University of Cambridge Cambridge UK; ^2^ Department of Biology Saint Francis University Loretto Pennsylvania USA; ^3^ Department of Biological Sciences University of Bristol Bristol UK; ^4^ Institute of Marine Science University of Auckland Auckland New Zealand; ^5^ School of Agriculture, Biomedicine and Environment La Trobe University Melbourne Australia

**Keywords:** dissection, invasive species, micro‐CT scanning, vocalisation

## Abstract

Vocal signalling is an important mode of communication in fishes. The two species of lionfish in the *Pterois* complex, the Indo‐Pacific lionfish (*Pterois volitans)* and the red lionfish (*Pterois miles*), are both known to produce different types of sounds with sonic muscles attached to the swimbladder. However, the specific mechanism and the functions of these vocalisations in these invasive species are still unknown. We used three‐dimensional bioimaging to describe the anatomy of the sonic muscles of both species. We further quantified the muscles of *P. volitans* to specifically explore how muscles developed across ontogeny and to test the hypothesis that sonic muscles would show sexual dimorphism if they were a sexually selected trait. Both *P. volitans* and *P. miles* showed a physoclistous swimbladder with a bilaterally symmetric pair of extrinsic sonic swimbladder muscles (ESSMs), which have been suggested to control buoyancy and generate vocalisations. Both species also displayed an additional pair of anterior extrinsic muscles, which projected dorsoventrally from the spinal column and inserted onto the anterior wall of the swimbladder, potentially also having a role in sound production. Both types of sonic muscles were present across ontogeny. Quantification of the posterior belly of the ESSMs in *P. volitans* showed that both the length and mass of these muscles in both mature and immature individuals increased linearly with body size. There were no ontogenetic or sex differences in sonic muscle investment between individuals. Given the primary function of these muscles is to control the swimbladder for buoyancy, this may constrain the modification of these muscles relative to body size, or they may have no differences in their acoustic function between sexes or across ontogeny.

## 1 INTRODUCTION

Acoustic signalling using vocalisations is an important mode of communication in fishes (Amorim, 2006). These signals serve social functions such as individual recognition (Myrberg & Riggio, 1985), competitor evaluation during aggressive interactions (Ladich, 1997) and courtship displays (Amorim et al., 2003; Dos Santos et al., 2000; Mann & Lobel, 1997; Miguel Simões et al., 2008). Vocalisations in fishes commonly comprise pulses of low frequency sounds with fundamental frequencies in the range 100–300 Hz depending on the species (Ladich, 2019), and can be produced in multiple ways. For example, some fishes use stridulation, by rubbing body parts together, like the grinding of pharyngeal teeth or bony structures against each other (Bertucci et al., 2014; Ladich, 2022). Another vocalisation mechanism includes vibrating the swimbladder (Fänge, 1966). Indeed, while the primary function of the swimbladder is for controlling buoyancy (Fänge, 1966; Parmentier et al., 2023), it is also involved in hearing and sound production (Popper & Fay, 2011; Popper & Schilt, 2008). Specifically, muscles that insert onto the swimbladder and contract the organ to alter its gas volume for buoyancy can also be vibrated against its wall to produce sounds (Dos Santos et al., 2000; Kasumyan, 2008; Tavolga, 1971). Some of these sonic muscles associated with the swimbladder in fishes are composed of the fastest vibrating striated muscle type in vertebrates and can reach contraction frequencies of up to 200 Hz, as seen in the oyster toadfish (*Opsanus tau*; Mok et al., 2011). Depending on the fish species, these muscles are thought to have evolved from occipital, epaxial, hypaxial or pectoral girdle muscles primarily involved in locomotion (Parmentier et al., 2017; Parmentier & Fine, 2016).

Different species of fishes have different vocal repertoires which vary in fundamental frequency, number, duration and rate of repetition of different sounds (Amorim, 2006). Theory predicts that in order to propagate and receive signals, sympatric sonic organisms should reduce acoustic competition by altering the characteristics or locations of their calls (Krause, 1993; Mullet et al., 2017). This results in the diversification of both acoustic signals and signalling behaviour. In closely related species, such variability in vocalisations, particularly those associated with courtship, could promote reproductive isolation (Amorim, 2006). For instance, hybrids of the cichlids *Chindongo saluosi* and *Maylandia estherae* produce courtship vocalisations with distinctly different duration and pulse period compared to either parent species, suggested to potentially promote speciation (Parmentier et al., 2024). Intraspecific variation in sound production is also common, with ontogenetic and sexual differences between individuals. For example, in the croaking gourami (*Trichopsis vittata*), sound pressure level, croak duration, number of pulses in a croak and pulse period increase with ontogeny, while dominant frequency decreases (Henglmüller & Ladichm, 1999). Moreover, juvenile grey gurnards (*Eutrigla gurnardus*) are more active sound producers than adults (Amorim & Hawkins, 2005). Differences in vocal behaviour can also result in anatomical dimorphism. For instance, male Lusitanian toadfish (*Halobactrachus didactylus*) possess larger swimbladders and sonic muscles than females, as males alone produce their signature ‘boat whistle’ mating calls (Dos Santos et al., 2000). Further, sneaker male plainfin midshipmen (*Porichthys notatus*) show six‐fold smaller sonic musculature relative to their smaller body sizes compared to nest‐guarding males (Brantley & Bass, 1994).

Two species of lionfish in the *Pterois* species complex (family: Scorpaenidae), *Pterois volitans* (Linnaeus, 1758) and *Pterois miles* (Bennett, 1828), have become established beyond their native ranges. Lionfish (*Pterois* spp.) were introduced into the Western Atlantic Ocean and Caribbean Sea in the 1980s and are now widely invasive (Albins & Hixon, 2013; Morris, 2009). More recently, *P. miles* traversed the Suez Canal as a Lessepsian migrant and established in the Mediterranean Sea (Turan et al., 2017). In both invaded ranges, densities have increased to levels more than 10 times higher than their native ranges (Darling et al., 2011; Kleitou et al., 2024; Kulbicki et al., 2012). Predation by lionfish poses a threat to native fish diversity and abundance (Albins, 2015; Côté et al., 2013; Green, Akins, Maljković, & Côté, 2012). In the Western Atlantic and Caribbean Sea, mitochondrial DNA suggests that the invasion comprises up to 90% *P. volitans* with the remaining 10% *P. miles* (Hamner et al., 2007). Genetic analysis suggests that the invasive *P. miles* lineage originated from the Indian Ocean, and *P. volitans* arose through hybridisation between the Indian Ocean *P. miles* and a Pacific Ocean lineage including *P. lunulata/russelii* (Wilcox et al., 2018). The two species are visually similar, with meristic fin ray counts required to discriminate between them (Schultz, 1986). In the Western Atlantic Ocean, both *Pterois* species occur sympatrically and have been found to hybridise (Whitaker & Janosik, 2020; Wilcox et al., 2018). Whitaker and Janosik (2020) suggest that both the numbers of *P. miles* and the levels of hybridisation in the Western Atlantic are potentially underreported due to a focus on only D‐loop mtDNA analyses and the presence of confounding pseudogenes. They concluded that all individuals sampled on the east coast of North Carolina, USA, were hybrids when sequenced using mtDNA in addition to nuclear markers. This suggests that hybrids are fertile and can backcross with parental lineages (Wilcox et al., 2018). The reproductive barriers between species where they occur sympatrically, if any, are not yet known.

Recently, the vocalisations of unidentified invasive lionfish (*Pterois* spp.) were recorded for the first time (Beattie et al., 2017), resulting in further investigations into their sound production (Parmentier et al., 2023; Schärer‐Umpierre et al., 2019). Lionfish vocalisations are primarily composed of ‘hums’ and ‘knocks’. Knocks are most common and consist of one to eight pulses, while hums contained continuous energy at relatively low peak frequencies of ~50–860 Hz (Beattie et al., 2017; Parmentier et al., 2023). An intermittent ‘purr’ of a constant tone ending in a slight down sweep has been associated with courtship (Schärer‐Umpierre et al., 2019). However, differences between the vocalisations of the two invasive species of lionfish has not yet been investigated. Lionfish possess a pair of extrinsic muscles which insert onto the swimbladder. The tetanisation of these muscles at 80 Hz suggests that they are able to produce fast contractions, and this in addition to their position makes them the most likely candidates for the production of the sounds recorded (Parmentier et al., 2023). While the function of these vocalisations is not yet fully resolved, Schärer‐Umpierre et al. (2019) reported observations of non‐antagonistic pairs of lionfish interacting in the wild while producing characteristic narrow bandwidth, long duration calls. They suggest, therefore, that lionfish vocalisations are likely to play a role in courtship. Other records of lionfish vocalisations have occurred when lionfish were being disturbed or agitated (Beattie et al., 2017; Parmentier et al., 2023). Moreover, the observation that these sounds are often paired with deliberate movement of the dorsal spines may suggest that they can serve as aposematic signals (Parmentier et al., 2023). When unagitated solitary and grouped lionfish produce vocalisations, these differ in frequency and pulse rate compared to agitated calls (Beattie et al., 2017).

Here, we first describe and compare the sonic swimbladder anatomy of both species in the invasive *Pterois* species complex (*P. volitans* and *P. miles*) using micro‐CT scans where divergence in sonic anatomy could predict differences in the structure of their vocalisations and function of their sonic behaviour. Second, we quantify how the investment in the mass and length of the sonic muscle in *P. volitans* individuals changed with ontogeny and sex. If the vocalisations of lionfish are related to courtship, we might expect some intraspecific differences in the investment in sonic musculature relative to body size between males and females, as well as ontogenetic changes in the development of the muscles as individuals grow from juveniles into sexually mature adults. Describing the anatomy of these two species of invasive lionfish and understanding the role that vocalisations might play in their reproductive ecology has implications for their management within invaded ranges.

## 2 METHODS

### 2.1 Micro‐CT scanning

#### 2.1.1 Pterois miles

One mature (total length [TL] 160.30 mm, sex unknown) and one immature lionfish (TL 35.04 mm) of the species *Pterois miles* were sourced through the Tropical Marine Centre to the University of Cambridge and humanely euthanised following the Schedule 1 protocol of the Animals (Scientific Procedures) Act 1986 of humane killing for fish (Animal Procedures Committee 2009) using an overdose of tricaine methanesulfonate (MS‐222; 300 mg/L) followed by exsanguination. Prior to fixation, the ventral abdomens of the mature individuals were slit to increase penetration of the fixative solution. To fix, lionfish were submerged in formalin (10%) for 3 days, then rinsed in water three times, with each rinse lasting 1 h. Lionfish were then immersion‐stained using phosphotungstic acid (PTA, 1%) for a total of 6 weeks. During the 6 weeks the solution was refreshed 10 times (approximately every 3–4 days). After the completion of the PTA staining, the lionfish were wrapped in bubble wrap and mounted in a PVC tube for scanning through the Cambridge Biotomography Centre. Scans were conducted using a Nikon XTEK H 225 ST high‐resolution MicroCT scanner which created a stack of 1999 images through a full 360° rotation of the specimens using an X‐ray energy of 200 kV and 230 μA with a voxel size of 106 μm^3^. The first scans were completed on 8 October 2021, but the two mature lionfish were not sufficiently stained, therefore we carefully removed the skin using a scalpel to increase solution penetration and re‐stained them in PTA, refreshing the solution every 3–4 days until scanning. The mature individuals were rescanned successfully on 12 January 2022 after a total of 159 days in the stain.

#### 2.1.2 Pterois volitans

One juvenile *Pterois volitans* (TL 39.11 mm, sex unknown) preserved in 10% formalin which was donated from a personal collection was transferred into a 1% PTA solution and the solution was refreshed weekly for 4 weeks. The lionfish was wrapped in bubble wrap and mounted in a PVC tube and scanned through the University of Cambridge Biotomography Centre on 12 July 2021. Scans were conducted using a Nikon XTEK H 225 ST high‐resolution MicroCT scanner which created a stack of 1999 images through a full 360° rotation of the specimens using an X‐ray energy of 135 kV and 160 μA with a voxel size of 38 μm^3^.

One adult lionfish (TL 215 mm, sex unknown) of the species *Pterois volitans* was ordered through Ocean Reefs Marine Aquariums, Australia, and euthanised following Schedule 1 procedure with an overdose of MS‐222 (300 mg/L) followed by spiking of the brain on 22 December 2023. The fish was immersion‐fixed in 4% paraformaldehyde for 4 weeks and then transferred into a water solution of PTA 1%. The PTA solution was refreshed every 8 weeks. The skin was removed from the fish and a slit was introduced in the ventral part of the abdomen to allow a better penetration of the staining agent. A successful scan was completed on 9 August 2024 after a total of 204 days in stain. Micro‐CT scanning was performed with a phoenix nanotom m (Waygate Technologies) operated using xs control and phoenix datos|x acquisition software (Waygate Technologies). The specimen was wrapped in bubble wrap and mounted in a PVC tube for scanning. The scan collected 1799 projections through a full 360° rotation of the specimen, using an X‐ray energy of 140 kV and 300 μA with a voxel size of 30 μm^3^ and a 0.5 mm aluminium filter. A summary table of all scanning parameters can be found in Table S1.

#### 2.1.3 Three‐dimensional reconstructions

Volume reconstruction of all micro‐CT data was performed using the phoenix datos|x reconstruction software (Waygate Technologies) applying an inline median filter and ROI filter during reconstruction. Segmentations of the spine, swimbladder and swimbladder‐associated muscles as well as the visualisations were done using ORS Dragonfly version 2022.2 software (Comet Technologies Canada Inc., software available at https://dragonfly.comet.tech/).

#### 2.1.4 Dissection and quantification of sonic musculature (*Pterois volitans* only)

**FIGURE 1:**
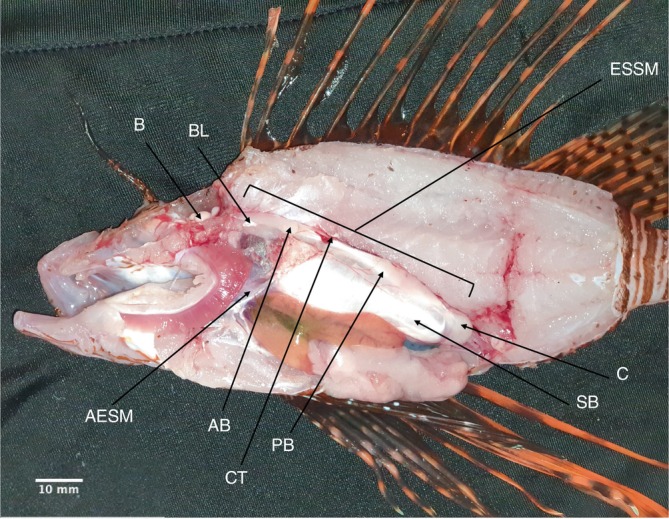
A cross‐section of an Indo‐pacific lionfish (*Pterois volitans*, TL 151.2 mm, sex unknown) showing (left to right, top to bottom) the brain (B), Baudelot's ligament (BL), extrinsic sonic swimbladder muscle (ESSM), the anterior extrinsic sonic muscle (AESM), the anterior belly of the extrinsic sonic swimbladder muscle (AB), the connective tissue between the bellies of the extrinsic sonic swimbladder muscle (CT), the posterior belly of the extrinsic sonic swimbladder muscle (PB), the swimbladder (SB) and the swimbladder cap (C), where the ESSM attaches posteriorly to the swimbladder.

Lionfish used in field studies conducted in Piscadera Bay in Curaçao, Dutch Caribbean (12°07′20.3″ N, 68°58′08.7″ W) in 2022 (Holmes, Hamilton, Simpson et al., 2025a) and 2023 (Holmes, Hamilton, Dunkly et al., 2025) were dissected following the end of behavioural experiments. Lionfish (*n* = 35) were caught over 6 weeks (March–June) in 2021 and *n* = 47 lionfish over 7 weeks (March–May) between April and May 2023. Fish were captured at dusk by SCUBA divers using clove oil and hand nets. Following capture, lionfish were brought up to a depth of 3 m at an ascent rate of ≤1 m min^−1^ with a 3‐min stop every 5 m to reduce the risk of barotrauma to the fish. Lionfish were kept individually in labelled cages made of PVC and wire mesh (30 × 30 cm) under a jetty in the ocean during experiments and then humanely euthanised following the Schedule 1 procedure of decapitation followed by destruction of the brain at the end of the experimental week in which they were used. Additional non‐experimental lionfish (*n* = 25) were donated by a lionfish hunter in 2021 for dissection. Lionfish were placed in a freezer at −18°C until dissection, which took place in June 2021 and May 2022. Freezing and thawing do not have a significant effect on the mass and density of muscles post‐mortem and should not affect any anatomical measurements taken (Leonard et al., 2022). During dissection, lionfish were first de‐spined and then dissected. The sex was determined by assessing the gonads of each individual using the guidelines proposed by Green, Akins, and Morris Jr. (2012). Individuals where muscles were damaged during dissection were discarded (*n* = 19). Individuals that were immature and for which sex could not be determined were recorded as ‘immature’. The sonic muscles associated with the swimbladder were removed and both the muscles and the lionfish were photographed next to a scale (*n* = 88 individuals in total). The TL of the individuals and the length of the sonic muscles, both total length where possible (*n* = 30), and posterior belly length (*n* = 80) were determined using Image J and the masses of both the total muscles (*n* = 30) and posterior belly of the sonic muscles (*n* = 88) were weighed on a Sartorius R 200 D micro‐balance scale. Since the wet weight of individuals was not taken, estimated mass based on the TL of the individuals was calculated using a length–weight relationship equation generated from 1991 lionfish collected in 2011 around Little Cayman in the Caribbean and ranging in TL from 27 to 391 mm by Edwards et al. (2014). Total wet weight (g) across both sexes and juveniles was calculated to be 0.3 × 10^−6^ × TL (mm)^3.24^. Individuals where sex was not determined (*n* = 1) or where there was an issue with the image preventing muscle length from being determined (*n* = 8) were removed from their respective analyses. To document the anatomy of the fish, one lionfish (*Pterois volitans*) was euthanised using an overdose of MS‐222 followed by confirmation of cessation of circulation following the Schedule 1 protocol and fully dissected and photographed (Figure 1) on 15 April 2022, but the muscles of this individual were not quantified nor was the sex determined.

#### 2.1.5 Ethical approval

All *Pterois miles* individuals used for micro‐CT scanning were euthanised under the University of Cambridge Animal Welfare and Ethical Review Body, application number 2021.5.18. The adult *Pterois volitans* used for micro‐CT scanning was euthanised under animal ethics application 2022/AE000569 UQ AEC NEWMA at the University of Queensland, Australia. In total, 82 of the 107 *Pterois volitans* individuals used for the quantification of sonic musculature were euthanised after being used in behavioural experiments that were approved by the University of Cambridge Animal Welfare and Ethical Review Body (UBS reference numbers OS2021/09 in 2022 and OS2022/14 in 2023). Lionfish are an invasive predator, representing a threat to local species and are therefore unable to be released following experiments. The remaining 25/107 *P. volitans* individuals were already dead when they were donated by a recreational lionfish hunter.

#### 2.1.6 Statistical analysis for the quantification of *Pterois volitans* sonic musculature

All statistical analyses were performed in R (Version 4.2.2, R Foundation for Statistical Computing, www.R-project.org). First, for those muscles where the majority of the extrinsic sonic swimbladder muscle (ESSM) was successfully dissected (*n* = 30/88), the full mass (g) and length (mm) of the muscles were modelled against the mass and length of only the posterior belly of the ESSM using linear regression. Since many of the muscles had damage at the point of connection to the skull, and the lengths and masses of the whole ESSMs were strongly correlated with those of the more accurately extracted posterior belly of the ESSM (muscle length: linear model [LM] *F*_1_ = 495.56, *r* ^2^ = 0.97, 95% confidence interval [CI] [1.54, 1.85], *p* < 0.001; muscle mass: LM *F*_1_ = 16.66, *r* ^2^ = 0.61, 95% CI [0.50, 1.49], *p* < 0.001), we used only the posterior belly for all further analyses. Effect sizes were calculated using Pearson's *r* ^2^ for models with a continuous predictor and Cohen's *D* for those with a categorical predictor. Model assumptions were assessed using the ‘DHARMa’ (Hartig & Hartig, 2017) and ‘effects’ (Fox, 2003; Fox & Weisberg, 2018) packages and additional analysis of non‐conformity was assessed using the ‘performance’ package (Lüdecke et al., 2021).

We first modelled how the mean length (mm) of the posterior belly of the ESSM scaled with body size (TL in mm), and the mean mass of the posterior belly of the ESSM scaled with estimated fish wet weight for each sex category (male, female and immature) using linear regressions. In the analysis of the male and the juvenile posterior ESSM mass as a function of estimated wet weight, outliers were identified that violated the model assumptions. We conducted analyses both with and without the outliers, and since the results did not qualitatively change, the outliers were retained.

The coefficient of allometry to quantify investment in the ESSMs when controlling for body size was calculated from the slope of linear regressions modelling the square‐root‐transformed posterior belly ESSM mass as a function of the square‐root‐transformed estimated wet weight, and the square‐root‐transformed posterior belly ESSM length and the square‐root‐transformed TL of the fish; values close to 1 represent isometry of the muscles, >1 represent hyper‐allometry and increased investment in sonic apparatus relative to body size and numbers <1 represent hypo‐allometry and relatively lower investment.

To analyse the mass and length of the ESSMs relative to the body size of the fish, sonic‐somatic indexes (SSIs) were calculated, similar to those used by Tellechea et al. (2022). This index helps to understand how the investment in sonic musculature varies in relation to body size, which can provide insights into the functional role of these muscles in sound production. SSI length was calculated as the mean posterior ESSM length (mm)/ L (mm) × 100, and SSI mass was calculated as mean posterior ESSM mass (g)/estimated wet weight (g) × 100. To determine whether there was an ontogenetic change in ESSM mass, we modelled the SSI length and SSI mass as a function of maturity (immature versus mature [male and female combined]). The model testing SSI mass as a function of maturity included an outlier that was retained in the analysis since its removal did not qualitatively affect the model outcome. To investigate differences in investment in the size (mass and length) of the posterior belly of the ESSM between mature male and female lionfish, we modelled the SSI length and SSI mass as a function of the sex of mature individuals only. There was one influential outlier in the model of SSI mass as a function of sex. When included, this model had a significant *p* value (LM: *F*_1_ = 4.51, *p* = 0.038). However, when removed, this result was no longer significant and therefore, to be conservative, the influential outlier was excluded from analysis. *F*‐tests on models with and without the factors of interest were conducted using the *drop1* function.

## 3 RESULTS

**FIGURE 2:**
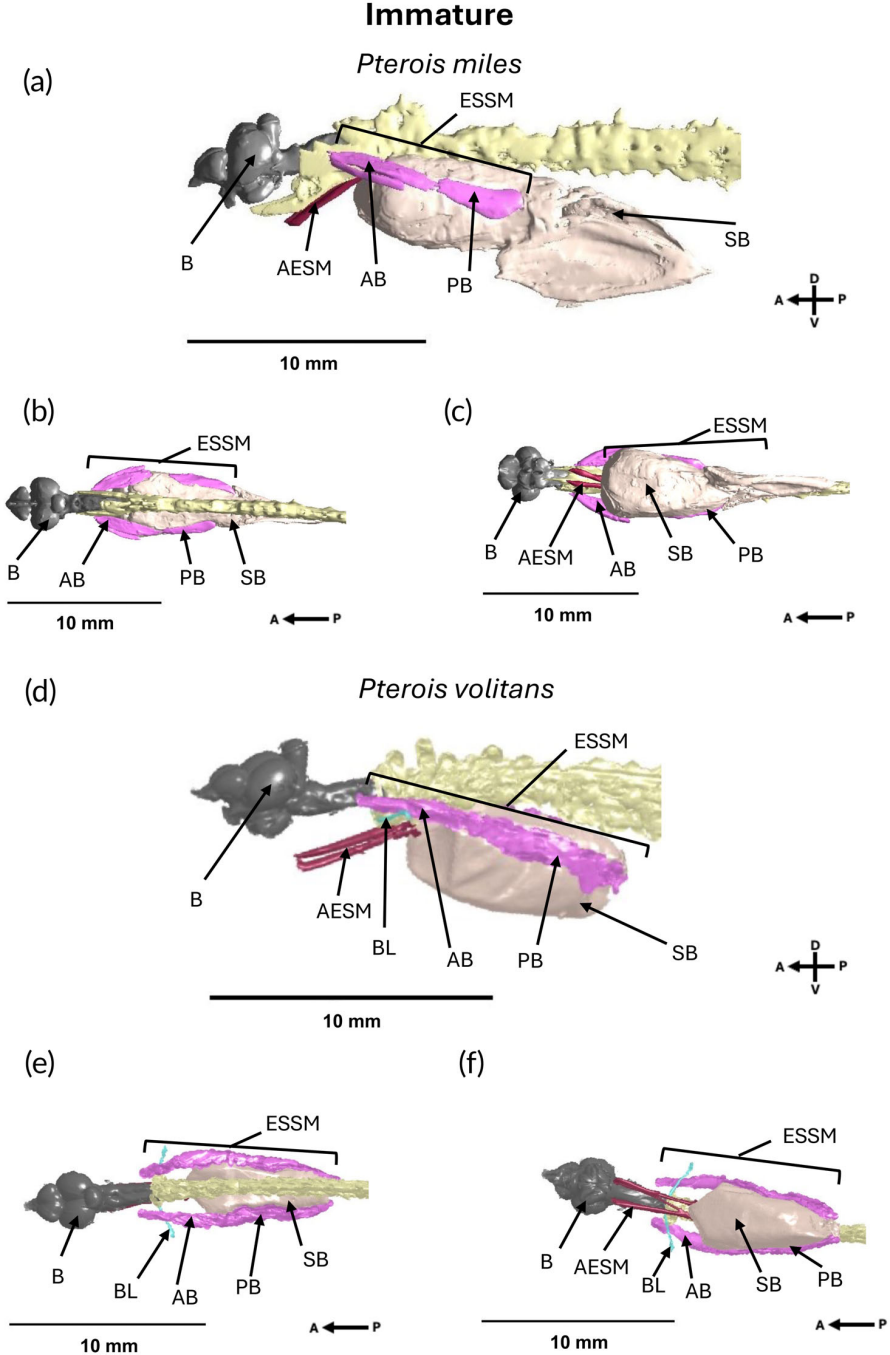
Micro‐CT scans of the (a) lateral, (b) dorsal and (c) ventral views of an immature *Pterois miles* (TL 35.04 mm, sex unknown) and the (d) lateral, (e) dorsal and (f) ventral views of an immature *Pterois volitans* (TL 39.11 mm, sex unknown) showing the brain (B), Baudelot's ligament (BL), extrinsic sonic swimbladder muscle (ESSM), the anterior extrinsic sonic muscle (AESM), the anterior belly of the extrinsic sonic swimbladder muscle (AB), the posterior belly of the extrinsic sonic swimbladder muscle (PB) and the swimbladder (SB). Axes show the anterior (A), posterior (P), dorsal (D) and ventral (V) orientation of the specimen.

**FIGURE 3:**
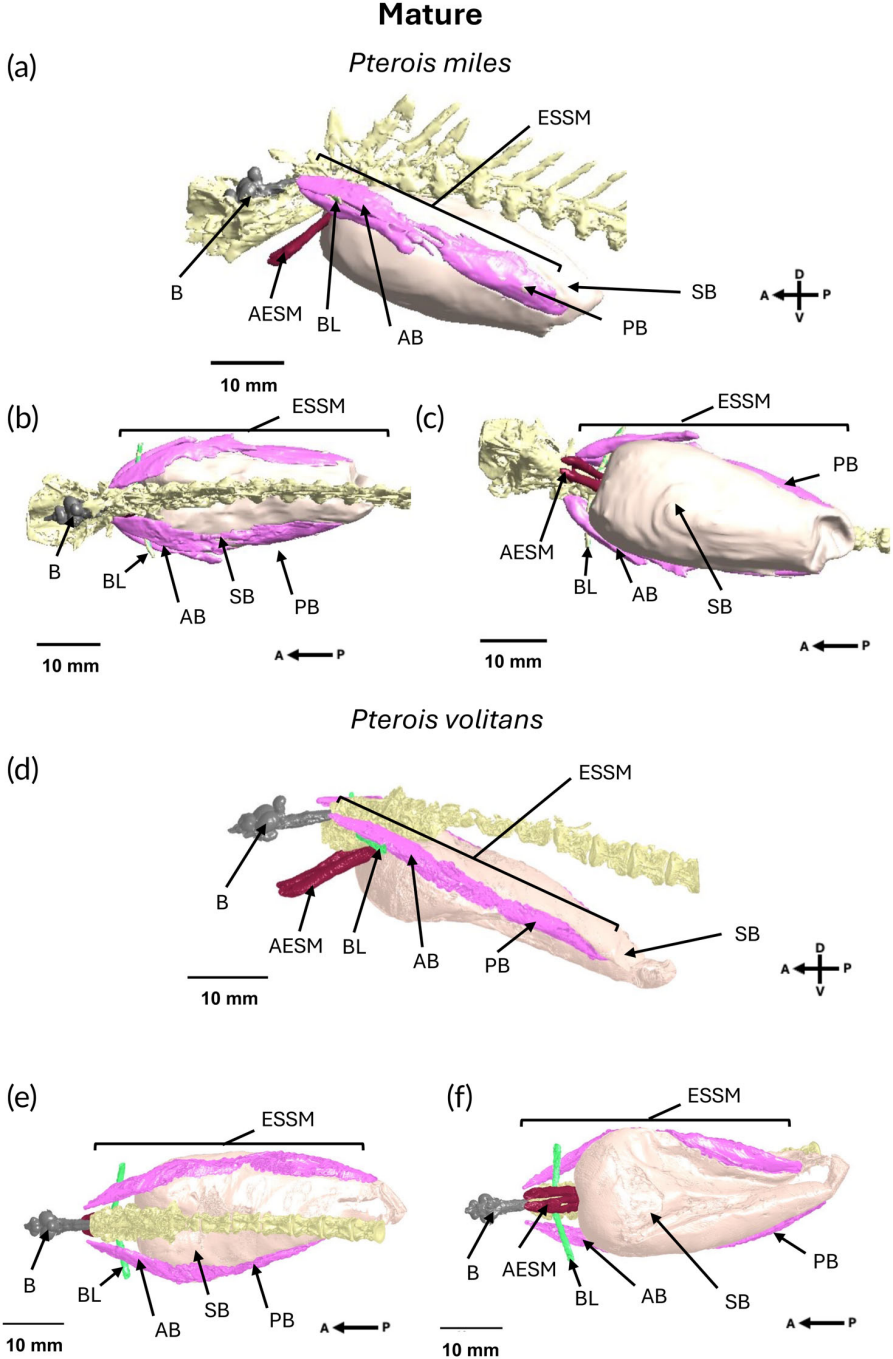
Micro‐CT scans of the (a) lateral, (b) dorsal and (c) ventral views of a mature *Pterois miles* (TL 160.30 mm, sex unknown) and the (d) lateral, (e) dorsal and (f) ventral views of a mature *Pterois volitans* (TL 215 mm, sex unknown) showing the brain (B), Baudelot's ligament (BL), extrinsic sonic swimbladder muscle (ESSM), the anterior extrinsic sonic muscle (AESM), the anterior belly of the extrinsic sonic swimbladder muscle (AB), the posterior belly of the extrinsic sonic swimbladder muscle (PB) and the swimbladder (SB). Axes show the anterior (A), posterior (P), dorsal (D) and ventral (V) orientation of the specimen.

*Pterois volitans* ESSMs connected from the exoccipital region of the skull, ran posteriorly dorsal to the Baudelot's ligament and terminated in the swimbladder cap at the posterior end of the swimbladder (Figures 1, 2, 3). We identified a further pair of anterior extrinsic sonic muscles (AESMs) that originated dorsally and extended downwards contacting the swimbladder, terminating at the lower jaw (Figures 1, 2, 3). The anterior insertion of these muscles onto the swimbladder makes them viable candidates as additional sonic muscles.

*Pterois miles* and *P. volitans* possessed similar musculature associated with the swimbladder. Both species had physoclistous‐type swimbladders and the same assembly of muscles associated with the swimbladder, all of which were visible in both immature (Figure 2) and mature individuals (Figure 3).

**FIGURE 4:**
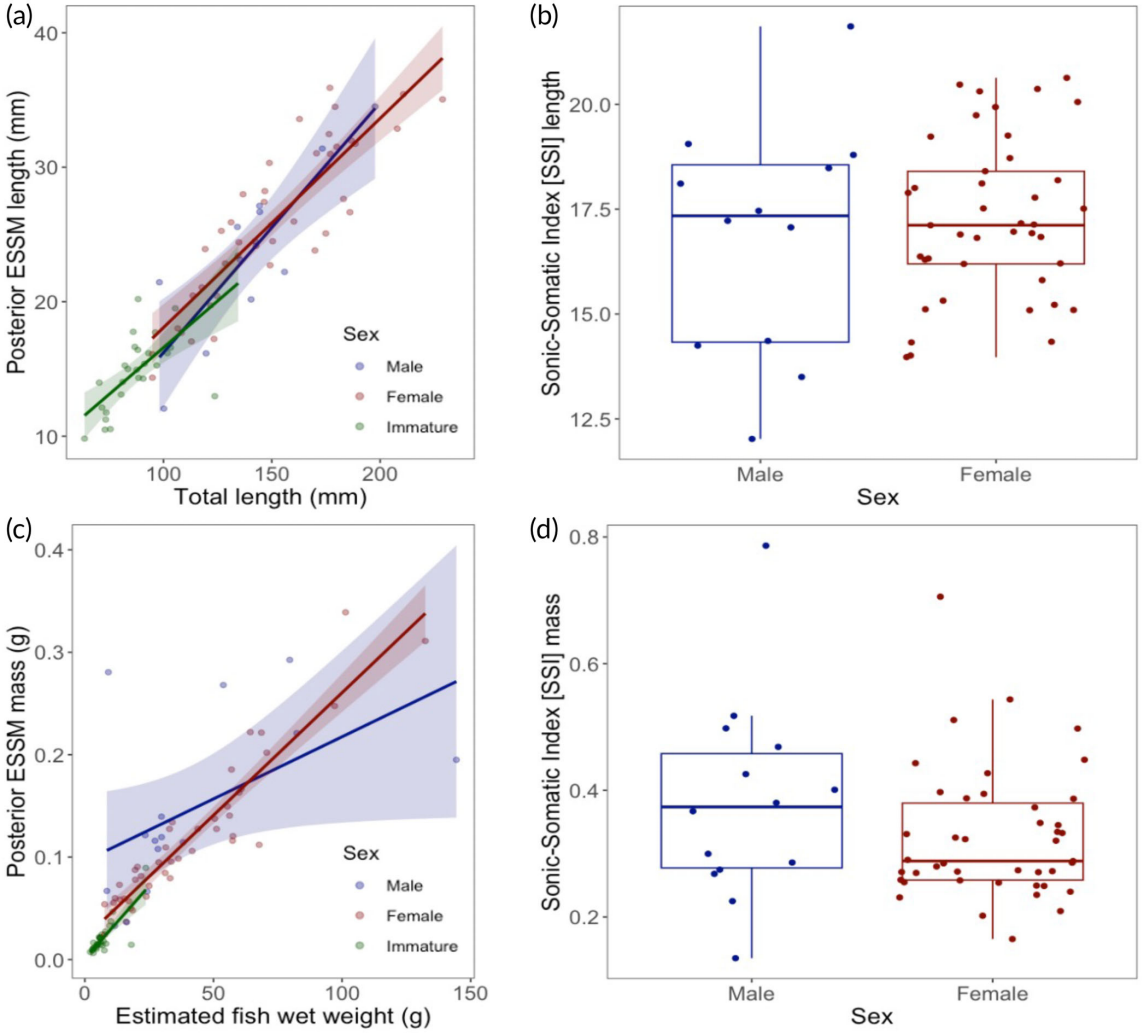
(a) The length of the posterior belly of the extrinsic sonic swimbladder muscle (ESSM) of Indo‐Pacific lionfish (*Pterois volitans*, *n* = 80) scaled linearly with body size (total length in mm) across ontogeny (linear model [LM]: male *F*_1_ = 24.96, *p* = 0.003; female *F*_1_ = 121.63, *p* < 0.001; immature *F*_1_ = 24.04, *p* < 0.001). (b) There was no significant difference in the sonic‐somatic index [SSI] length (ESSM length (mm)/TL (mm) × 100) between the sexes in mature individuals (LM: *F*_1_ = 0.12, *p* = 0.73). (c) The posterior belly ESSM mass also scaled linearly with estimated fish wet weight across ontogeny (*n* = 88, LM: male: *F*_1_ = 4.69, *p* = 0.0495; female: *F*_1_ = 283.69, *p* < 0.001; immature: *F*_1_ = 48.25, *p* < 0.001) and (d) SSI mass (ESSM mass (g)/estimated wet weight (g) × 100) also did not differ between the sexes in mature individuals (LM: *F*_1_ = 1.29, *p* = 0.26). The shaded area represents 95% confidence intervals, boxplots show the median and 1st and 3rd interquartile ranges and whiskers represent 95% confidence intervals.

There was a linear relationship between the length of the posterior belly of the ESSM and fish total length in both mature male and female *Pterois volitans* lionfish (LM: male *F*_1_ = 24.96, *r* ^2^ = 0.84, 95% CI [0.10, 0.27], *p* = 0.003; female *F*_1_ = 121.63, *r* ^2^ = 0.87, 95% CI [0.13, 0.18], *p* < 0.001; Figure 4a), as well as in immature individuals (immature *F*_1_ = 24.04, *r* ^2^ = 0.70, 95% CI [0.08, 0.20], *p* < 0.001; Figure 4a). Males had a mean posterior ESSM length of 23.74 ± 1.77, females a mean of 26.14 ± 0.92 and immature individuals had a mean of 15.00 ± 0.60 (Table 1). All individuals showed near isometric investment in posterior belly ESSM muscle length (length coefficients of allometry: overall = 1.01, male = 1.14, female = 0.96, immature = 0.88; Table 1). The mass of the posterior belly of the ESSM also had a linear relationship with estimated fish wet weight (LM: male *F*_1_ = 4.69, *r* ^2^ = 0.51, 95% CI [2.93e–06, 2.42e–03], *p* = 0.0495; female *F*_1_ = 283.69, *r* ^2^ = 0.93, 95% CI [0.0021,0.0027], *p* < 0.001; immature *F*_1_ = 48.25, *r* ^2^ = 0.80, 95% CI [0.0020, 0.0037], *p* < 0.001; Figure 4b) but displayed hypo‐allometric investment in mass with growth (mass coefficients of allometry: overall = 0.92, male = 0.49, female = 0.74, immature = 0.81), with males possessing a mean posterior ESSM mass of 0.145 ± 0.22, females a mean mass of 0.118 ± 0.011 and immature individuals a mean mass of 0.020 ± 0.0029 (Table 1).

**TABLE 1:** A summary of the anatomical measurements of and quantities of *Pterois volitans* lionfish.

Maturity	Sex	*n*	Total length (mm) ± SE	Est. body mass (g) ± SE	Posterior ESSM length (mm) ± SE	Posterior ESSM mass (g) ± SE	Sonic somatic index length ± SE	Sonic somatic index mass ± SE	Coefficient of allometry: length	Coefficient of Allometry: mass
All	All	88	129.36 ± 4.38	28.92 ± 3.09	22.021 ± 0.81	0.089 ± 0.0084	17.11 ± 0.24	0.36 ± 0.033	1.01	0.92
Immature	–	30	88.44 ± 2.75	6.76 ± 0.81	15.00 ± 0.60	0.020 ± 0.0029	16.99 ± 0.45	0.31 ± 0.016	0.89	0.81
Mature	All	58	150.53 ± 4.41	40.39 ± 3.90	25.60 ± 0.82	0.125 ± 0.0098	17.17 ± 0.29	0.39 ± 0.050	1.005	0.66
	Male	15	148.29 ± 9.92	40.45 ± 9.47	23.74 ± 1.77	0.145 ± 0.22	16.85 ± 0.81	0.56 ± 0.18	1.14	0.49
	Female	43	151.31 ± 4.91	40.36 ± 4.17	26.14 ± 0.92	0.118 ± 0.011	17.26 ± 0.30	0.33 ± 0.016	0.96	0.74

Abbreviations: ESSM, extrinsic sonic swimbladder muscle; SE, standard error.

There was no ontogenetic difference in the SSI length of *P. volitans* posterior belly ESSMs between mature (male and female combined) and immature individuals (LM: *F*_1_ = 0.12, *d* = 0.081, 95% CI [−0.85, 1.21], *p* = 0.73) with a mean SSI length of 17.17 ± 0.29 for mature individuals and 16.99 ± 0.45 for immature ones (Table 1). Additionally, there was no difference in the SSI mass index of the posterior belly ESSM across ontogeny (LM: *F*_1_ = 1.29, *d* = 0.26, 95% CI [−0.60, 0.22], *p* = 0.26) with a mean SSI mass of 0.39 ± 0.050 for mature and 0.31 ± 0.016 for immature individuals respectively (Table 1). Further, when looking at mature individuals only, there was also no difference in either the SSI length or SSI mass between males and females (LM: SSI length: *F*_1_ = 0.35, *d* = 0.19, 95% CI [−0.99, 1.81], *p* = 0.56; LM: SSI mass: *F*_1_ = 2.20, *d* = 0.46, 95% CI [−0.13, 0.019], *p* = 0.14), whereby males had a mean SSI length of 16.85 ± 0.81 and SSI mass of 0.56 ± 0.18, while females had an SSI length of 17.26 ± 0.30 and an SSI mass of 0.33 ± 0.016 (Table 1).

## 4 DISCUSSION

The invasive Indo‐Pacific lionfish (*Pterois volitans*) and the Indian Ocean red lionfish (*Pterois miles*) possessed similar sonic anatomy. Both had a bilaterally symmetric pair of extrinsic sonic swimbladder muscles (ESSMs) that originated at the occipital region of the skull. These muscles ran dorsal to the Baudelot's ligament, inserted laterally onto a physoclistous swimbladder and terminated at the swimbladder cap at the posterior end of the swimbladder. The ESSM was made up of two muscle lobes, termed ‘bellies’, joined by connective tissue. Both species also had an additional pair of anterior extrinsic sonic muscles (AESMs) that projected dorsoventrally from the spinal column and inserted onto the anterior wall of the swimbladder. The positioning of these muscles suggests that they may also be able to generate vocalisations. Both types of sonic muscles were visible across ontogeny. Quantification of the posterior belly of the ESSM in *P. volitans* showed that the length of the sonic muscles of individuals scaled with TL and had isometric investment across ontogeny, while the mass showed hypo‐allometric investment relative to body size. There was no ontogenetic or sex differences in investment in the posterior belly with muscle mass and length scaling isometrically with estimated wet weight and TL of individuals.

Lionfish vocalisations are generally composed of low frequency ‘knocks’, ‘pulses’ and ‘hums’ (Beattie et al., 2017; Parmentier et al., 2023; Schärer‐Umpierre et al., 2019). Hums are generally produced through vibration of the extrinsic sonic swimbladder muscle against the lateral walls of the swimbladder (Connaughton et al., 2000; Ladich & Fine, 2006) and possess a mean peak frequency of 53 Hz and duration of 2220 ms (Parmentier et al., 2023), while knock‐type sound pulses are thought to be generated by rapid twitches in the sonic muscles (Connaughton et al., 2000). Pulses have a mean period of 19.5 Hz and knocks have a peak frequency of 115 Hz and last for 83 ms (Parmentier et al., 2023). The previously unreported pair of AESMs that inserted onto the anterior wall of the swimbladder are similar in location to the ventral muscles identified in male cryptic cusk eels (*Parophidion vassali*), which possess three pairs of sonic muscles. These fishes possess the unusual ability to produce sounds starting with low‐amplitude pulses followed by high‐amplitude pulses, although cusk eels also possess additional adaptations such as wing‐like processes on the vertebra and an osseus swimbladder plate for sound production (Parmentier et al., 2022). While Parmentier et al. (2023) did not fully elucidate the alternance of pulses of high and low amplitude in lionfishes, our suggestion is that these second pair of muscles likely contribute to vocal production. Whether these muscles are the superfast striated sonic muscles usually associated with vocalisations is unknown. A secondary type of sonic muscle termed ‘slow muscles’ is found in carapid fishes that are able to produce sounds (Parmentier et al., 2006). Confirming whether these muscles are capable of generating sounds, and the mechanism by which they do so, would require quantifying the twitch parameters of the muscles and the frequency at which they tetanise.

A purported function of the vocalisations in lionfish is for sexual reproduction, either through male–male competition or through male–female displays. Indeed, the ‘purr’ vocalisations reported by Schärer‐Umpierre et al. (2019) were recorded during non‐agonistic behavioural displays between pairs of lionfish, anticipated to be a reproductive pair due to the size difference between individuals. It is perhaps surprising, therefore, that we did not detect any differences in the mass or length of the sonic muscles between immature and mature individuals, or between males and females. This absence of differences between males and females could result from our catches not coinciding with spawning season because male muscle status can vary temporally. For example, in the vocalising black drum (*Pogonias courbina*), during the spawning season, androgens drive hypertrophy of the sonic muscles, followed by rapid post‐spawning atrophy (Tellechea et al., 2022). This pattern is also seen in the weakfish (*C. regalis*; Connaughton et al., 2000) and the fawn cusk eel (*Lepophidium profundorum*; Nguyen et al., 2008). This explanation, however, is unlikely as lionfish are capable of year‐round reproduction (Fogg et al., 2017; Morris, 2009). Moreover, in Little Cayman in the Caribbean, seasonality in gonadosomatic indices in female lionfish has been observed and was most pronounced during March/April and August (Gardner et al., 2015), the former which coincides with the timing of our sampling. However, this directly contradicts histology on individuals from the Gulf of Mexico, where males were found to be spawning capable in all months except March, when no actively spawning females were present (Fogg et al., 2017). The high variability in ESSM mass in male lionfish could also suggest that males might undergo asynchronous hypertrophy and atrophy in their sonic muscles following spawning events to mitigate any costs associated with larger sonic muscles. Temporal measurements of lionfish sonic muscles would therefore be worthwhile.

An alternative reason why we could not detect differences in sonic muscle mass and length between males and females could reflect a smaller portion of individuals in the study being sexually active. Histology on lionfishes in Bermuda suggested that the minimum reproductive size of lionfish is >189 cm (Eddy et al., 2019). In our study, only six individuals had reached this size, with individuals in our study having a mean total length of 129 mm, despite showing sexual characteristics necessary for determining the sex. This could also explain the variability seen in the posterior belly ESSM masses of the larger individuals, where only a portion of the largest individuals in this study may have reached sexual maturity. Therefore, only a small proportion of individuals in our study may have been actively using their muscles for vocal courtship. Alternatively, the high variation in muscle mass and length of males and females and lack of statistical differences could be an artefact of the lower number of larger individuals sampled overall, resulting in limited statistical power to detect differences between the sexes. Finally, Parmentier et al. (2023) observed that stimulation of the posterior belly of the ESSM resulted in shortening of the swimbladder, while the same was not true of stimulating the anterior belly. They concluded that the anterior belly is responsible for vocalisation while the posterior belly for buoyancy. Sexual dimorphism in courting males may mean that hypertrophy of the sonic muscles would only be visible in the anterior belly of the ESSM, which we were unable to successfully extract in a sufficient number of samples.

Another explanation for a lack of differences between sexes in our measures of posterior belly ESSM length and mass could be that males and females do not differ in the frequency and/or duration of vocalisations. The muscles associated with the swimbladder serve two functions: while they are involved in the production of vocalisations, first and foremost they are used to control buoyancy (Parmentier et al., 2023). This dual functionality may constrain the development of the sonic muscles. Lionfish locomotion is complex, involving high manoeuvrability and individuals adopting a wide range of body pitches when stalking or striking prey (Parmentier et al., 2023). While some fishes, for example the Lusitanian toadfish (*Halobatrachus didactylus*), are able to maintain sexual dimorphism in swimbladder and sonic muscle size, the toadfish is sedentary and benthic (Pereira et al., 2021) and may have reduced requirements on the swimbladder for buoyancy compared to lionfish. The rapid atrophy of male sonic muscles in toadfish post‐spawning suggests a cost associated with maintaining increased sonic muscle mass (Tellechea et al., 2022). This cost may be prohibitive for lionfish to develop since reproduction is not restricted to an isolated spawning season.

The function of vocalisations in both *P. volitans* and *P. miles* could instead be restricted to agonistic interactions. This is supported by multiple records of lionfish vocalisations occurring when lionfish were being disturbed or agitated (Beattie et al., 2017; Parmentier et al., 2023). Other Scorpaenid fishes, such as the marbled rockfish (*Sebasticus marmoratus*), produce vocalisations during agonistic interactions (Nichols, 2005). Vocalisation can be particularly important in territorial species. For example, vocalising male bicolour damselfish (*Pomacentrus partitus*) that hold territories are more likely to maintain their territory and prevent intrusion when actively vocalising (Myrberg, 1997). Vocalisations may serve as honest signals during agonistic and dominance interactions since dominant frequency has a direct relationship with fish size in many fish species (Amorim, 2006) and can predict the outcome of contests. For example, in croaking gouramis (*Trichopsis vittata*), the lower dominant frequency and higher sound pressure level of croaks during agonistic displays predicted success and dominance status (Ladich, 1998). Lionfish generally show high site‐fidelity, with tagged individuals being caught within limited home ranges (Jud & Layman, 2012), although some individuals can move larger distances (Tamburello & Côté, 2015). Overt agonism between individuals has not, however, been observed in *Pterois* lionfish (Tamburello & Côté, 2015), which are frequently found aggregated (Hunt et al., 2019) and share refugia (García‐Rivas et al., 2017). Given that agonistic behaviour in *Pterois* lionfishes has not yet been fully described, this does not negate the possibility that agonistic displays between lionfish are purely sonic and lack a visual component. However, in response to an approaching rod in tanks, lionfish produce ‘hums’ that are paired with erected fins in the direction of the rod (Parmentier et al., 2023). These displays could occur as an aposomatic display in response to a perceived predator and not have a function in interactions between conspecifics. This, however, seems unlikely, since similar hum‐type vocalisations are also recorded when lionfish are together with other lionfish (Beattie et al., 2017).

Vocalisations during agonistic interactions may have a role across various stages of development. Our finding that the sonic muscles were developed early and scaled with body size suggests a role of vocalisation across ontogeny, likely during agonistic interactions. For example, grey gurnards (*Eutrigla gurnardus*) produce agonistic vocalisations during feeding both as juveniles and as adults (Amorim & Hawkins, 2005). The hypo‐allometry in posterior belly ESSM muscle mass relative to body size across ontogeny could suggest that while lionfish do produce vocalisations, their importance does not warrant disproportionately high investment with growth. This could indicate that while the muscles are necessary for sound production, high investment could be limited by functional constraints related to buoyancy or other physiological factors. The larynx in mammals also has dual functionality, serving as an organ for both breathing and vocalisation. In rat lines bred to select for pups to produce either lower or higher frequency calls to signal their affective state, individuals which produced higher rates of high frequency sounds displayed shorter vocal folds and more mineralised thyroid cartilage compared to those who produced fewer ultrasonic calls (Lesch et al., 2021). This shows that while dually functional vocal apparatus may be constrained by their primary function, selection can still act to create divergence in vocal anatomy as a consequence of differing vocal behaviours (Lesch et al., 2021). Alternatively, invasion can result in reduced agonism, as is seen between individuals in some species such as Argentine ants (*Linepithema humile*; Holway et al., 1998), which in turn could result in atrophy of the muscles or selection for individuals with relatively smaller sonic muscle investment. A quantitative comparison of the sonic musculature of lionfishes between their invaded and native ranges would, therefore, be worthwhile.

The similarity in sonic muscle assemblage associated with the swimbladder between the two *Pterois* lionfishes is likely due to the close relationship between the sister groups and only shallow genetic divergence (Whitaker & Janosik, 2020; Wilcox et al., 2018). Due to the similarities of their appearance, *P. volitans* and *P. miles* were initially considered synonymous (de Beaufort, 1962; Dor, 1984) and were only later described as two distinct species (Schultz, 1986). Given the similarity in sonic musculature, species in the *Pterois* complex are likely capable of creating similar types of sounds, but their vocal repertoire and the function of vocalisations within their ecology may differ. For example, five species of Malawi cichlids in the *Pseudotropheus zebra* complex produce distinctly different courtship signals differing in the number of pulses and pulse period despite having undergone rapid adaptive radiation and therefore being likely to possess similar vocal muscles (Amorim et al., 2008). The largest divergence in the acoustic variables within vocalisations occurred between the sympatric cichlid species. Moreover, the same muscles can produce sounds of different frequencies. For example, in the weakfish (*Cynoscion regalis*), the frequency of a sound pulse is determined by the rate of muscle contraction rather than the size of the swimbladder (Connaughton et al., 2000). Indeed, despite producing different sounds, three species of Doradid catfish did not possess differences in swimbladder size, muscle anatomy or muscle length (Boyle et al., 2015). Instead, differences in sound production were attributed to differences in neural activation of the sonic muscles. It would be valuable, therefore, to record the vocal repertoire of the different lionfish species paired with behavioural observations, particularly in areas where both species occur sympatrically. If vocalisations are species specific, this would allow for passive acoustic monitoring to be used for discerning between the visually cryptic species. Moreover, leveraging of playback experiments would also be useful for determining if divergence in repertoire creates reproductive barriers between these species. For example, in closely related species of grasshopper mice (*Onychomys Arenicola* and *O. torridus*), vocalisations differed in fundamental frequency in sympatry but not in allopatry (Campbell et al., 2019). One theory about the success of *P. volitans* in the western Atlantic is that the hybridisation between *P. miles* and *P. russelii/lunulata* to create *P. volitans* conferred some level of heterosis (i.e. hybrid vigour; Wilcox et al., 2018). If introgression does indeed increase the fitness of invasive individuals, then an understanding of the reproductive barriers or lack thereof between these species in their invaded range and the level of hybridisation warrants further investigation.

Many of the efforts to control lionfish populations involve manual removal by human hunters (Andradi‐Brown, 2019), which is time‐consuming, expensive and limited by recreational SCUBA diver limits, causing deeper mesophotic reefs to act as a sink of large fecund individuals (Andradi‐Brown et al., 2017). This has led to the development and deployment of unmanned traps that can operate at deeper depths (Gittings et al., 2017; Harris et al., 2019, 2020). Adding sounds as an attractant has been suggested as a means to increase the efficacy of unmanned traps (Harris et al., 2020). Therefore, a greater understanding of the role of vocalisation in the ecology of invasive lionfish could help guide the selection of suitably attractive acoustic stimuli to improve trap design and support lionfish management efforts. Alternatively, if lionfish vocalisations are utilised during agonistic interactions, this could suggest that broadcasting agonistic vocalisations could be used in control efforts to influence lionfish behaviour.

## AUTHOR CONTRIBUTIONS

This study was conceived by R.B.H. and L.C., and designed by R.B.H., L.C. and J.H.‐R. Preparation of specimens was conducted by R.B.H., K.E.C. and L.C. Scanning and segmentation were completed by K.Z.S. and L.C. Data collection was performed by R.B.H. and N.M.H. Data preparation and statistical analysis were performed by R.B.H. and J.H.‐R. The manuscript was written by R.B.H., and all authors were involved in editing the manuscript.

## CONFLICT OF INTEREST STATEMENT

We declare no conflicts of interest.

## Supporting information


**TABLE S1.** Scanning parameters for micro‐CT scans.
**TABLE S2.** Model outputs from analyses of the sonic swimbladder muscle anatomy of Indo‐Pacific lionfish (*Pterois volitans*). Significant results are highlighted in bold and asterisks denote the level of significance.

## Data Availability

The raw data and R scripts that support the findings of this study are available in Figshare with the identifier DOI: https://doi.org/10.6084/m9.figshare.28007858.

## References

[jfb70183-bib-0001] Albins, M. A. (2015). Invasive Pacific lionfish *Pterois volitans* reduce abundance and species richness of native Bahamian coral‐reef fishes. Marine Ecology Progress Series, 522, 231–243. 10.3354/meps11159

[jfb70183-bib-0002] Albins, M. A. , & Hixon, M. A. (2013). Worst case scenario: Potential long‐term effects of invasive predatory lionfish (*Pterois volitans*) on Atlantic and Caribbean coral‐reef communities. Environmental Biology of Fishes, 96, 1151–1157. 10.1007/s10641-011-9795-1

[jfb70183-bib-0003] Amorim, M. C. P. (2006). Diversity of sound production in fish. Communication in Fishes, 1, 71–104.

[jfb70183-bib-0005] Amorim, M. C. P. , & Hawkins, A. D. (2005). Ontogeny of acoustic and feeding behaviour in the Grey gurnard, *Eutrigla gurnardus* . Ethology, 111, 255–269. 10.1111/j.1439-0310.2004.01061.x

[jfb70183-bib-0004] Amorim, M. C. P. , Fonseca, P. J. , & Almada, V. C. (2003). Sound production during courtship and spawning of *Oreochromis mossambicus*: Male–female and male–male interactions. Journal of Fish Biology, 62, 658–672. 10.1046/j.1095-8649.2003.00054.x

[jfb70183-bib-0006] Amorim, M. C. P. , Simões, J. M. , Fonseca, P. J. , & Turner, G. F. (2008). Species differences in courtship acoustic signals among five Lake Malawi cichlid species (*Pseudotropheus* spp.). Journal of Fish Biology, 72, 1355–1368. 10.1111/j.1095-8649.2008.01802.x

[jfb70183-bib-0007] Andradi‐Brown, D. A. (2019). Invasive lionfish (*Pterois volitans* and *P. miles*): Distribution, impact, and management. In Y. Loya , K. Puglise , & T. Bridge (Eds.), Mesophotic coral ecosystems. Coral reefs of the world (Vol. 12). Springer. 10.1007/978-3-319-92735-0_48

[jfb70183-bib-0008] Andradi‐Brown, D. A. , Vermeij, M. J. , Slattery, M. , Lesser, M. , Bejarano, I. , Appeldoorn, R. , Goodbody‐Gringley, G. , Chequer, A. D. , Pitt, J. M. , Eddy, C. , & Smith, S. R. (2017). Large‐scale invasion of western Atlantic mesophotic reefs by lionfish potentially undermines culling‐based management. Biological Invasions, 19, 939–954. 10.1007/s10530-016-1358-0

[jfb70183-bib-0009] Beattie, M. , Nowacek, D. P. , Bogdanoff, A. K. , Akins, L. , & Morris, J. A. (2017). The roar of the lionfishes *Pterois volitans* and *Pterois miles* . Journal of Fish Biology, 90, 2488–2495. 10.1111/jfb.13321 28470766

[jfb70183-bib-0010] Bertucci, F. , Ruppé, L. , van Wassenbergh, S. , Compère, P. , & Parmentier, E. (2014). New insights into the role of the pharyngeal jaw apparatus in the sound‐producing mechanism of *Haemulon flavolineatum* (Haemulidae). Journal of Experimental Biology, 217, 3862–3869. 10.1242/jeb.109025 25355850

[jfb70183-bib-0011] Boyle, K. S. , Riepe, S. , Bolen, G. , & Parmentier, E. (2015). Variation in swim bladder drumming sounds from three doradid catfish species with similar sonic morphologies. Journal of Experimental Biology, 218(18), 2881–2891. 10.1242/jeb.123414 26206358

[jfb70183-bib-0012] Brantley, R. K. , & Bass, A. H. (1994). Alternative male spawning tactics and acoustic signals in the Plainfin midshipman fish *Porichthys notatus* Girard (Teleostei, Batrachoididae). Ethology, 96, 213–232. 10.1111/j.1439-0310.1994.tb01011.x

[jfb70183-bib-0013] Campbell, P. , Arévalo, L. , Martin, H. , Chen, C. , Sun, S. , Rowe, A. H. , Webster, M. S. , Searle, J. B. , & Pasch, B. (2019). Vocal divergence is concordant with genomic evidence for strong reproductive isolation in grasshopper mice (Onychomys). Ecology and Evolution, 9, 12886–12896. 10.1002/ece3.5770 31788222 PMC6875671

[jfb70183-bib-0014] Connaughton, M. A. , Taylor, M. H. , & Fine, M. L. (2000). Effects of fish size and temperature on weakfish disturbance calls: Implications for the mechanism of sound generation. Journal of Experimental Biology, 203, 1503–1512. 10.1242/jeb.203.9.1503 10751166

[jfb70183-bib-0015] Côté, I. M. , Green, S. J. , & Hixon, M. A. (2013). Predatory fish invaders: Insights from indo‐Pacific lionfish in the western Atlantic and Caribbean. Biological Conservation, 50, 50–64. 10.1016/j.biocon.2013.04.014

[jfb70183-bib-0016] Darling, E. S. , Green, S. J. , O'Leary, J. K. , & Côté, I. M. (2011). Indo‐Pacific lionfish are larger and more abundant on invaded reefs: A comparison of Kenyan and Bahamian lionfish populations. Biological Invasions, 13, 2045–2051. 10.1007/S10530-011-0020-0/FIGURES/2

[jfb70183-bib-0017] de Beaufort, L. F. (1962). The fishes of the indo‐Australian archipelago. 10.1163/9789004590953

[jfb70183-bib-0018] Dor, M. (1984). Checklist of the fishes of the Red Sea. https://agris.fao.org/search/en/providers/122621/records/647396c368b4c299a3fb70a0

[jfb70183-bib-0019] Dos Santos, M. E. , Modesto, T. , Matos, R. J. , Grober, M. S. , Oliveira, R. F. , & Canário, A. (2000). Sound production by the Lusitanian toad fish *Halobatrachus didactylus* . Bioacoustics, 10, 309–321. 10.1080/09524622.2000.9753440

[jfb70183-bib-0020] Eddy, C. , Pitt, J. , Oliveira, K. , Morris, J. A. , Potts, J. , & Bernal, D. (2019). The life history characteristics of invasive lionfish (*Pterois volitans* and *P. miles*) in Bermuda. Environmental Biology of Fishes, 102, 887–900. 10.1007/s10641-019-00877-4

[jfb70183-bib-0021] Edwards, M. A. , Frazer, T. K. , & Jacoby, C. A. (2014). Age and growth of invasive lionfish (*Pterois* spp.) in the Caribbean Sea, with implications for management. Bulletin of Marine Science, 90, 953–966. 10.5343/bms.2014.1022

[jfb70183-bib-0022] Fänge, R. (1966). Physiology of the swimbladder. Physiological Reviews, 46, 299–322. 10.1152/physrev.1966.46.2.299 5325971

[jfb70183-bib-0023] Fogg, A. Q. , Brown‐Peterson, N. J. , & Peterson, M. S. (2017). Reproductive life history characteristics of invasive red lionfish (*Pterois volitans*) in the northern Gulf of Mexico. Bulletin of Marine Science, 93, 791–813. 10.5343/bms.2016.1095

[jfb70183-bib-0076] Fox, J. (2003). Effect displays in R for generalised linear models. Journal of statistical software, 8, 1–27.

[jfb70183-bib-0077] Fox, J. , & Weisberg, S. (2018). Visualizing fit and lack of fit in complex regression models with predictor effect plots and partial residuals. Journal of statistical software, 87, 1–27.

[jfb70183-bib-0024] García‐Rivas, M. D. C. , Machkour‐M'Rabet, S. , Pérez‐Lachaud, G. , Schmitter‐Soto, J. J. , Doneys, C. , St‐Jean, N. , Cobián, D. , & Hénaut, Y. (2017). What are the characteristics of lionfish and other fishes that influence their association in diurnal refuges? Marine Biology Research, 13, 899–908. 10.1080/17451000.2017.1314496

[jfb70183-bib-0025] Gardner, P. G. , Frazer, T. K. , Jacoby, C. A. , & Yanong, R. P. E. (2015). Reproductive biology of invasive lionfish (*Pterois* spp.). Frontiers in Marine Science, 2, 7. 10.3389/fmars.2015.00007

[jfb70183-bib-0026] Gittings, S. R. , Fogg, A. Q. , Frank, S. , Hart, J. V. , Clark, A. , Clark, B. , Noakes, S. E. , & Fortner, R. L. (2017). Going deep for lionfish: Designs for two new traps for capturing lionfish in deep water. https://repository.library.noaa.gov/view/noaa/30773

[jfb70183-bib-0028] Green, S. J. , Akins, J. L. , & Morris, J. A., Jr. (2012). Lionfish dissection: Techniques and applications NOAA technical memorandum NOS NCCOS 139. https://repository.library.noaa.gov/view/noaa/719

[jfb70183-bib-0027] Green, S. J. , Akins, J. L. , Maljković, A. , & Côté, I. M. (2012). Invasive Lionfish Drive Atlantic Coral Reef Fish Declines. PLoS One, 7, e32596. 10.1371/journal.pone.0032596 22412895 PMC3296711

[jfb70183-bib-0029] Hamner, R. M. , Freshwater, D. W. , & Whitfield, P. E. (2007). Mitochondrial cytochrome b analysis reveals two invasive lionfish species with strong founder effects in the western Atlantic. Journal of Fish Biology, 71, 214–222. 10.1111/j.1095-8649.2007.01575.x

[jfb70183-bib-0030] Harris, H. E. , Fogg, A. Q. , Gittings, S. R. , Ahrens, R. N. M. , Allen, M. S. , & Patterson, W. F. (2020). Testing the efficacy of lionfish traps in the northern Gulf of Mexico. PLoS One, 15, e0230985. 10.1371/journal.pone.0230985 32845879 PMC7449463

[jfb70183-bib-0031] Harris, H. E. , Patterson, W. F. , Ahrens, R. N. M. , & Allen, M. S. (2019). Detection and removal efficiency of invasive lionfish in the northern Gulf of Mexico. Fisheries Research, 213, 22–32. 10.1016/j.fishres.2019.01.002

[jfb70183-bib-0075] Hartig, F. , & Hartig, M. F. (2017). Package ‘dharma’. R package, 531, 532.

[jfb70183-bib-0032] Henglmüller, S. M. , & Ladichm, F. (1999). Development of agonistic behaviour and vocalization in croaking gouramis. Journal of Fish Biology, 54, 380–395. 10.1111/j.1095-8649.1999.tb00837.x

[jfb70183-bib-0074] Holmes, R. B. , Hamilton, N. M. , Dunkley, K. , & Herbert‐Read, J. E. (2025). Lionfish (Pterois volitans) show social attraction to conspecifics when selecting shelters. Ethology, e13575.

[jfb70183-bib-0033] Holmes, R. B. , Hamilton, N. M. , Simpson, S. D. , & Herbert‐Read, J. E. (2025a). Lionfish (*Pterois volitans*) do not show directional preferences for ambient underwater soundscapes during diurnal hours. Marine Biology, 172, 9. 10.1007/s00227-024-04554-8

[jfb70183-bib-0034] Holway, D. A. , Suarez, A. V. , & Case, T. J. (1998). Loss of intraspecific aggression in the success of a widespread invasive social insect. Science, 282, 949–952. 10.1126/science.282.5390.949 9794767

[jfb70183-bib-0035] Hunt, C. L. , Kelly, G. R. , Windmill, H. , Curtis‐Quick, J. , Conlon, H. , Bodmer, M. D. V. , Rogers, A. D. , & Exton, D. A. (2019). Aggregating behaviour in invasive Caribbean lionfish is driven by habitat complexity. Scientific Reports, 9, 1–9. 10.1038/s41598-018-37459-w 30692608 PMC6349842

[jfb70183-bib-0036] Jud, Z. R. , & Layman, C. A. (2012). Site fidelity and movement patterns of invasive lionfish, *Pterois* spp., in a Florida estuary. Journal of Experimental Marine Biology and Ecology, 414, 69–74. 10.1016/j.jembe.2012.01.015

[jfb70183-bib-0037] Kasumyan, A. O. (2008). Sounds and sound production in fishes. Journal of Ichthyology, 48, 981–1030. 10.1134/s0032945208110039

[jfb70183-bib-0038] Kleitou, P. , Rees, S. E. , Kletou, D. , Harris, H. E. , Cai, L. L. , Green, S. , Hadjioannou, L. , Savva, I. , Giovos, I. , Jimenez, C. , & Hall‐Spencer, J. M. (2024). Marine protected areas can increase the abundance of invasive lionfish (*Pterois miles*). Conservation Science and Practice, 6, e13147. 10.1111/csp2.13147

[jfb70183-bib-0039] Krause, B. L. (1993). The niche hypothesis: A virtual symphony of animal sounds, the origins of musical expression and the health of habitats. The Soundscape Newsletter, 6, 4–6.

[jfb70183-bib-0040] Kulbicki, M. , Beets, J. , Chabanet, P. , Cure, K. , Darling, E. , Floeter, S. R. , Galzin, R. , Green, A. , Harmelin‐Vivien, M. , Hixon, M. , Letourneur, Y. , de Loma, T. L. , McClanahan, T. , McIlwain, J. , MouTham, G. , Myers, R. , O'Leary, J. K. , Planes, S. , Vigliola, L. , & Wantiez, L. (2012). Distributions of indo‐Pacific lionfishes *Pterois* spp. in their native ranges: Implications for the Atlantic invasion. Marine Ecology Progress Series, 446, 189–205. 10.3354/MEPS09442

[jfb70183-bib-0041] Ladich, F. (1997). Agonistic behaviour and significance of sounds in vocalizing fish. Marine and Freshwater Behaviour and Physiology, 29, 87–108. 10.1080/10236249709379002

[jfb70183-bib-0042] Ladich, F. (1998). Sound characteristics and outcome of contests in male croaking Gouramis (Teleostei). Ethology, 104, 517–529. 10.1111/j.1439-0310.1998.tb00087.x

[jfb70183-bib-0043] Ladich, F. (2019). Ecology of sound communication in fishes. Fish and Fisheries, 20, 552–563. 10.1111/FAF.12368 31130820 PMC6519373

[jfb70183-bib-0044] Ladich, F. (2022). Shut up or shout loudly: Predation threat and sound production in fishes. Fish and Fisheries, 23, 227–238. 10.1111/faf.12612

[jfb70183-bib-0045] Ladich, F. , & Fine, M. L. (2006). Sound‐generating mechanisms in fishes: A unique diversity in vertebrates. Communication in Fishes, 1, 3–43.

[jfb70183-bib-0046] Leonard, K. C. , Worden, N. , Boettcher, M. L. , Dickinson, E. , & Hartstone‐Rose, A. (2022). Effects of freezing and short‐term fixation on muscle mass, volume, and density. Anatomical Record, 305, 199–208. 10.1002/ar.24639 33843149

[jfb70183-bib-0047] Lesch, R. , Schwaha, T. , Orozco, A. , Shilling, M. , Brunelli, S. , Hofer, M. , Bowling, D. L. , Zimmerberg, B. , & Fitch, W. T. (2021). Selection on vocal output affects laryngeal morphology in rats. Journal of Anatomy, 238(5), 1179–1190. 10.1111/joa.13366 33480050 PMC8053590

[jfb70183-bib-0078] Lüdecke, D. , Ben‐Shachar, M. S. , Patil, I. , Waggoner, P. , & Makowski, D. (2021). Performance: An R package for assessment, comparison and testing of statistical models. Journal of Open Source Software, 6, 3139.

[jfb70183-bib-0048] Mann, D. A. , & Lobel, P. S. (1997). Propagation of damselfish (Pomacentridae) courtship sounds. The Journal of the Acoustical Society of America, 101, 3783–3791. 10.1121/1.418425

[jfb70183-bib-0049] Miguel Simões, J. , Duarte, I. G. , Fonseca, P. J. , Turner, G. F. , & Clara Amorim, M. (2008). Courtship and agonistic sounds by the cichlid fish *Pseudotropheus zebra* . The Journal of the Acoustical Society of America, 124, 1332–1338. 10.1121/1.2945712 18681618

[jfb70183-bib-0050] Mok, H.‐K. , Parmentier, E. , Chiu, K.‐H. , Tsai, K.‐E. , Chiu, P.‐H. , & Fine, M. L. (2011). An intermediate in the evolution of superfast sonic muscles. Frontiers in Zoology, 8, 31. 10.1186/1742-9994-8-31 22126599 PMC3251524

[jfb70183-bib-0051] Morris, J. A. (2009). The biology and ecology of the invasive indo‐Pacific lionfish. North Carolina State University.

[jfb70183-bib-0052] Mullet, T. C. , Farina, A. , & Gage, S. H. (2017). The acoustic habitat hypothesis: An Ecoacoustics perspective on species habitat selection. Biosemiotics, 10, 319–336. 10.1007/s12304-017-9288-5

[jfb70183-bib-0053] Myrberg, A. A. (1997). Sound production by a coral reef fish (*Pomacentrus partitus*): Evidence for a vocal, territorial ‘keep‐out’ signal. Bulletin of Marine Science, 60, 1017–1025.

[jfb70183-bib-0054] Myrberg, A. A. , & Riggio, R. J. (1985). Acoustically mediated individual recognition by a coral reef fish (*Pomacentrus partitus*). Animal Behaviour, 33, 411–416. 10.1016/S0003-3472(85)80065-8

[jfb70183-bib-0055] Nguyen, T. K. , Lin, H. , Parmentier, E. , & Fine, M. L. (2008). Seasonal variation in sonic muscles in the fawn cusk‐eel *Lepophidium profundorum* . Biology Letters, 4, 707–710. 10.1098/rsbl.2008.0383 18812307 PMC2614160

[jfb70183-bib-0056] Nichols, B. (2005). Characterizing sound production in nearshore rockfishes (*Sebastes* spp.) [Doctoral dissertation] University of South Florida.

[jfb70183-bib-0058] Parmentier, E. , & Fine, M. L. (2016). Fish sound production: Insights. In R. A. Suthers , W. T. Fitch , R. R. Fay , & A. N. Popper (Eds.), Vertebrate sound production and acoustic communication (pp. 19–49). Springer International Publishing. 10.1007/978-3-319-27721-9_2

[jfb70183-bib-0057] Parmentier, E. , Diogo, R. , & Fine, M. L. (2017). Multiple exaptations leading to fish sound production. Fish and Fisheries, 18, 958–966. 10.1111/faf.12217

[jfb70183-bib-0059] Parmentier, E. , Gaëlle, S. , Renaud, B. , Fine, M. L. , Loïc, K. , Lucia, D. I. , & Marta, B. (2022). Sound production and mechanism in the cryptic cusk‐eel *Parophidion vassali* . Journal of Anatomy, 241, 581–600. 10.1111/joa.13691 35666031 PMC9358751

[jfb70183-bib-0060] Parmentier, E. , Herrel, A. , Banse, M. , Hornstra, H. , Bertucci, F. , & Lecchini, D. (2023). Diving into dual functionality: Swim bladder muscles in lionfish for buoyancy and sonic capabilities. Journal of Anatomy, 244, 1–259. 10.1111/JOA.13963 37891703 PMC10780155

[jfb70183-bib-0061] Parmentier, E. , Lagardère, J.‐P. , Braquegnier, J.‐B. , Vandewalle, P. , & Fine, M. L. (2006). Sound production mechanism in carapid fish: First example with a slow sonic muscle. Journal of Experimental Biology, 209, 2952–2960. 10.1242/jeb.02350 16857879

[jfb70183-bib-0062] Parmentier, E. , Raick, X. , Leblanc, N. , Santoni‐Guichard, G. , Banse, M. , de Jean Dieu Da, S. , & van Damme, A. (2024). Acoustic innovations in courtship sounds generated by hybridization in cichlid fish. Zoological Journal of the Linnean Society, 200, 755–762. 10.1093/zoolinnean/zlad094

[jfb70183-bib-0063] Pereira, T. J. , Almeida, P. R. , Quintella, B. R. , Gronningsaeter, A. , Costa, M. J. , Marques, J. P. , & Costa, J. L. (2021). Fine‐scale behaviour of the Lusitanian toadfish assessed in situ with the AccelTag. Animal Biotelemetry, 9, 10. 10.1186/s40317-021-00231-5

[jfb70183-bib-0064] Popper, A. N. , & Fay, R. R. (2011). Rethinking sound detection by fishes. Hearing Research, 273, 25–36. 10.1016/j.heares.2009.12.023 20034550

[jfb70183-bib-0065] Popper, A. N. , & Schilt, C. R. (2008). Hearing and acoustic behavior: Basic and applied considerations. In J. F. Webb , R. R. Fay , & A. N. Popper (Eds.), Fish Bioacoustics. Springer Handbook of Auditory Research (Vol. 32). Springer. 10.1007/978-0-387-73029-5_2

[jfb70183-bib-0066] Schärer‐Umpierre, M. T. , Zayas, C. , Appeldoorn, R. S. , Tuohy, E. , Olson, J. C. , Keller, J. A. , & Acosta, A. (2019). The purr of the lionfish: Sound and behavioral context of wild lionfish in the greater Caribbean. Gulf and Caribbean Research, 30, GCFI15–GCFI19. 10.18785/gcr.3001.17

[jfb70183-bib-0067] Schultz, E. T. (1986). *Pterois volitans* and *Pterois miles*: Two valid species. Copeia, 1986, 686–690. 10.2307/1444950

[jfb70183-bib-0068] Tamburello, N. , & Côté, I. M. (2015). Movement ecology of indo‐Pacific lionfish on Caribbean coral reefs and its implications for invasion dynamics. Biological Invasions, 17, 1639–1653. 10.1007/s10530-014-0822-y

[jfb70183-bib-0069] Tavolga, W. N. (1971). Sound production and detection (Vol. 5, pp. 135–205). Fish Physiology. 10.1016/S1546-5098(08)60047-3

[jfb70183-bib-0070] Tellechea, J. S. , Izquierdo, S. , Perez, W. , & Norbis, W. (2022). Sound variation by hypertrophy and atrophy sonic muscle in the male southern black drum (*Pogonias courbina*). The Journal of the Acoustical Society of America, 152, 429–436. 10.1121/10.0012690 35931508

[jfb70183-bib-0071] Turan, C. , Uygur, N. , & İğde, M. (2017). Lionfishes *Pterois miles* and *Pterois volitans* in the north‐eastern Mediterranean Sea: Distribution, habitation, predation and predators. Natural and Engineering Sciences, 2, 35–42. 10.28978/nesciences.292355

[jfb70183-bib-0072] Whitaker, J. M. , & Janosik, A. M. (2020). Missing the mark(er): Pseudogenes identified through whole mitochondrial genome sequencing provide new insight into invasive lionfish genetics. Conservation Genetics, 21, 467–480. 10.1007/s10592-020-01263-9

[jfb70183-bib-0073] Wilcox, C. L. , Motomura, H. , Matsunuma, M. , & Bowen, B. W. (2018). Phylogeography of lionfishes (*Pterois*) indicate taxonomic over splitting and hybrid origin of the invasive *Pterois volitans* . Journal of Heredity, 109, 162–175. 10.1093/jhered/esx056 28637254

