# Supplementary material for: Structural and developmental insights into the muscles involved in lionfish (Pterois spp.) vocalisations
Source: J Fish Biol. 2025 Aug 28;107(6):1982–94. doi: 10.1111/jfb.70183 (PMC12861829; doi:10.1111/jfb.70183)
Supplement: Supplementary file 1 — TABLE S1. Scanning parameters for micro‐CT scans. TABLE S2. Model outputs from analyses of the sonic swimbladder muscle anatomy of Indo‐Pacific lionfish (Pterois volitans). Significant results are highlighted in bold and asterisks denote the level of significance. [file JFB-107-1982-s001.docx]

Structural and developmental insights into sonic muscles in invasive lionfish (*Pterois* spp.)

Roxanne B Holmes^1^, Nadia M Hamilton^1^, Katharine E Criswell^2^, Keturah Z Smithson^1^, James E Herbert-Read^1,3^ and Lucille Chapuis^4,5^

^1^ Department of Zoology, University of Cambridge, Cambridge, UK

^2^ Department of Biology, Saint Francis University, USA

^3^ Department of Biology, Lund University, Lund, Sweden

^4^ Department of Biological Sciences, University of Bristol, UK

^5^ Institute of Marine Science, University of Auckland, NZ

**Supplementary material**

Table S1: Scanning parameters for the micro-CT scans

| **Species** | **Maturity** | **Stack size** | **Voxel size (μm^3^)** | **kV** | **μA** |
| --- | --- | --- | --- | --- | --- |
| *Pterois miles* | Immature | 1999 | 106 | 200 | 230 |
|  | Mature | 1999 | 106 | 200 | 230 |
| *Pterois volitans* | Immature | 1999 | 38 | 135 | 160 |
|  | Mature | 1799 | 30 | 140 | 300 |

Table S2: Model outputs from analyses of the sonic swimbladder muscle anatomy of Indo-Pacific lionfish (*Pterois volitans*). Significant results are highlighted in **bold** and asterisks denote level of significance.

| **Analysis** | **Model** | **AIC** | **Fixed factor** | **Estimate** | **Effect size** | **95% CI** | **F_1_** | **p** |
| --- | --- | --- | --- | --- | --- | --- | --- | --- |
| **Total ESSM length ~ posterior ESSM length** | Linear regression | 141.74 | Posterior belly length | 1.69 | r^2^ = 0.97 | 1.54, 1.85 | 495.56 | **< 0.001***** |
| **Total ESSM mass ~**  **posterior ESSM mass** | Linear regression | -55.92 | Posterior belly mass | 0.99 | r^2^ = 0.61 | 0.50, 1.49 | 16.66 | **< 0.001***** |
| **Posterior belly length (Male) ~ TL** | Linear regression | 67.52 | TL | 0.19 | r^2^ = 0.84 | 0.10, 0.27 | 24.96 | **< 0.001***** |
| **Posterior belly length (Female) ~ TL** | Linear regression | 208.34 | TL | 0.16 | r^2^ = 0.87 | 0.13, 0.18 | 121.63 | **<0.001***** |
| **Posterior belly length (Immature) ~ TL** | Linear regression | 125.02 | TL | 0.14 | r^2^ = 0.70 | 0.08, 0.20 | 24.04 | **< 0.001***** |
| **Posterior belly mass (Male) ~ Est. wet weight** | Linear regression | -30.56 | Estimated wet weight | 0.001 | r^2^ = 0.51 | 2.93e-06, 2.42e-03 | 4.69 | **0.0495*** |
| **Posterior belly mass (Female) ~ Est. wet weight** | Linear regression | -190.62 | Estimated wet weight | 0.002 | r^2^ = 0.93 | 0.0021, 0.0027 | 283.59 | **< 0.001***** |
| **Posterior belly mass (Immature) ~ Est. wet weight** | Linear regression | -188.55 | Estimated wet weight | 0.003 | r^2^ = 0.80 | 0.0020, 0.0037 | 48.25 | **< 0.001***** |
| **SSI length ~ Maturity** | Linear regression | 356.46 | Maturity | 0.18 | d = 0.081 | -0.85, 1.21 | 0.12 | 0.73 |
| **SSI mass ~ Maturity** | Linear regression | 49.11 | Maturity | 0.08 | d = 0.26 | -0.60, 0.22 | 1.29 | 0.26 |
| **SSI length ~ Sex**  **(Mature only)** | Linear regression | 234.21 | Sex | 0.41 | d = 0.19 | -0.99, 1.81 | 0.35 | 0.56 |
| **SSI mass ~ Sex**  **(Mature only)** | Linear regression | 52.51 | Sex | -0.23 | d = 0.64 | -0.46, -0.013 | 4.51 | **0.038*** |
| **SSI mass ~ Sex**  **(Mature only)** | Linear regression (influential outlier removed) | -76.67 | Sex | -0.054 | d = 0.46 | -0.13, 0.019 | 2.20 | 0.14 |
